# Comprehensive Analysis of the Expression and Prognostic Value of SPINT1/2 in Breast Carcinoma

**DOI:** 10.3389/fendo.2021.665666

**Published:** 2021-07-26

**Authors:** Qiulin Wu, Guobing Yin, Jing Luo, Yingzi Zhang, Tiantian Ai, Jiao Tian, Yudi Jin, Jinwei Lei, Shengchun Liu

**Affiliations:** ^1^ Department of Endocrine and Breast Surgery, The First Affiliated Hospital of Chongqing Medical University, Chongqing, China; ^2^ Department of Breast and Thyroid Surgery, Second Affiliated Hospital of Chongqing Medical University, Chongqing, China; ^3^ Department of Pathology, Chongqing Medical University, Chongqing, China; ^4^ Department of Cardiovascular Sciences, Chongqing Kangxin Hospital, Chongqing, China

**Keywords:** SPINT1, SPINT2, prognosis, functions, breast cancer

## Abstract

**Background:**

Hepatocyte growth factor (HGF) signaling plays a plethora of roles in tumorigenesis and progression in many cancer types. As HGF activator inhibitors, serine protease inhibitor, Kunitz types 1 and 2 (SPINT1 and SPINT2) have been reported to be differentially expressed in breast cancer, but their prognostic significance and functioning mechanism remain unclear.

**Methods:**

In our study, multiple databases and bioinformatics tools were used to investigate SPINT1/2 expression profiles, prognostic significance, genetic alteration, methylation, and regulatory network in breast carcinoma.

**Results:**

SPINT1/2 expression was upregulated in breast cancer, and was relatively higher in human epidermal growth factor receptor 2 (HER2) and node positive patients. Elevated SPINT1/2 expression was significantly correlated with a poorer prognosis. Genetic alterations and SPINT1/2 hypomethylation were observed. In breast carcinoma, SPINT1/2 were reciprocally correlated and shared common co-expressed genes. Gene ontology (GO) and the Kyoto Encyclopedia of Genes and Genomes (KEGG) enrichment analysis showed that their common co-expressed genes were primarily involved in regulating cell attachment and migration.

**Conclusions:**

Our study identified the expression profiles, prognostic significance and potential roles of SPINT1/2 in breast carcinoma. These study results showed that the SPINT1/2 were potential prognostic biomarker for patients with breast cancer.

## Introduction

Breast carcinoma is the most common malignancy and the second leading cause of cancer-related mortality in women worldwide (1). According to statistics published in 2020, breast cancer alone accounted for approximately 30% of all new cancer cases and 15% of all cancer-related deaths in women in the United States (1). Despite advances in early diagnosis and treatment, almost 5–10% of patients have metastatic lesions when diagnosed with breast cancer, of which only 20% can survive over 5 years (2). As a disease with high heterogeneity, current methods of prognostic prediction and management are still sub-optimal. Therefore, it is crucial to identify novel reliable prognostic biomarkers and treatment targets.

Signaling transduction by HGF and mesenchymal–epithelial transition tyrosine kinase receptor (MET) is aberrantly activated in many types of cancers, and experimental evidence suggests that the activation of the HGF/MET pathway facilitates cancer cell proliferation, therapy resistance, metastasis, and adaptive response to adverse microenvironments (3–5). HGF, a paracrine factor in the extracellular matrix, is activated by serine proteases mediated proteolysis after being synthesized and secreted by stromal cells as an inactive precursor, proHGF (6). HGF activator (HGFA) and matriptase, two dominant activators of proHGF, can be blocked by several endogenous inhibitors, particularly, SPINT1 and SPINT2 (7). These two protease inhibitors have been reported to be aberrantly expressed in many types of cancer and represent one of the mechanisms of HGF/MET signaling.

Previous studies have shown that HGF and MET expression are elevated and correlated with progression and poorer prognosis in breast cancer patients (8–10). Ectopic expression of SPINT1 or SPINT2 in fibroblasts induced a reduction in HGF levels, thus ablating the HGF-mediated metastatic influence on MDA-MB-231 breast cancer cells (11). Therefore, SPINT1/2 were theoretically supposed to be downregulated in breast carcinoma. However, Parr et al. reported that HGF and MET expression and the HGFA, SPINT1, and SPINT2 levels were relatively higher in breast cancer tissues by immunohistochemical investigations (12). Currently, there are few studies on the expression and functions of SPINT1 and SPINT2 in breast cancer. As crucial regulators of a key transmembrane signaling in mammary malignancies, SPINT1 and SPINT2 should be scrutinized thoroughly. This study aimed to investigate the expression and roles of SPINT1/2 in breast cancer using bioinformatics approaches.

## Materials and Methods

### Analysis of The SPINT1/2 Expression

The expression of SPINT1/2 was examined with Oncomine, the Cancer Genome Atlas (TCGA), and the Human Protein Atlas (HPA) databases. The Oncomine database (https://www.oncomine.org/resource/login.html) consists of abundant microarray data across 35 cancer types and advanced analytical tools to facilitate data mining in cancer research (13). The inclusion threshold for the published SPINT1/2 expression datasets was as follows: *P <*1E−4, fold change higher or less than 2, and gene rank of the top 10%. Moreover, HTseq-FPKM data for breast cancer samples were downloaded from TCGA (https://portal.gdc.cancer.gov/) and converted to TMP with R package ‘zFPKM’, Those data were arranged using R and normalized with ‘DESeq2’ package. The differentially expressed genes were identified using ‘limma’ package. The differential expression of SPINT1/2 and hub genes were visualized with ‘ggplot2’. We analyzed the protein expression of SPINT1/2 in normal and breast cancer tissues using HPA database (https://www.proteinatlas.org/), which contains a large compendium of transcriptomic data and over 10 million images showing immunohistochemistry and immunocytochemistry staining of human proteins spanning 17 cancer types (14). SPINT1/2 expression profiles in cancer patients with different molecular subtypes and node status were explored with Breast Cancer Gene-Expression Miner v4.5 (http://bcgenex.centregauducheau.fr/) (15, 16).

### Patients and Samples

A total of 21 human breast cancer specimens, including 8 HER2+ and 13 HER2- samples, were obtained from the First Affiliated Hospital of Chongqing Medical University. All patients (41–72 years old) underwent mammary resection for breast cancer at the First Affiliated Hospital of Chongqing Medical University between May 2020 and May 2021. The status of hormone receptors and HER2 were determined according to the results of immunohistochemistry (IHC) by the Department of Pathology of Chongqing Medical University. The study was approved by the Ethics Committee of Chongqing Medical University. Written informed consent was obtained from all patients.

### IHC

The samples were fixed with 4% formaldehyde buffer. Deparaffinized tissues were then sectioned to into 4-µm-thick slices. Following antigen repair and endogenous peroxidase blocking, the sections were incubated with specific rabbit primary antibodies against SPINT1 (1:100; cat. no. FNab08182; FineTest) and SPINT2 (1:400; cat. no. bs-10062R; Bioss) overnight at 4°C. Next, the slices were treated with HRP-conjugated goat anti-rabbit IgG secondary antibody (1:300; cat. no. TA140003; OriGene) for 30 min at room temperature. Protein expression was detected with 3,3′-diaminobenzidine (OriGene) and hematoxylin staining and images were captured under Nikon Eclipse 80i microscope (magnification, ×200; Nikon Corporation). The mean optical density (MOD) in five randomly selected areas was calculated with Image-Pro Plus 6.0 software (Media Cybernetics, Inc.). SPINT1 and SPINT2 staining intensities (I) were scored as: 0 (no staining), 1 (weak staining), 2 (intermediate staining), 3 (strong staining) and 4 (very strong staining). The percentage of the positively stained area (A) was scored as: 1 (0–25%), 2 (26–50%), 3 (51–75%) and 4 (76–100%). The results were scored by adding up the intensity and percentage scores (I + A).

### Analysis of The Prognostic Significance of SPINT1/2

The prognostic significance of SPINT1/2 was evaluated using Kaplan–Meier plotter and PrognoScan. The Kaplan–Meier plotter database (https://kmplot.com/analysis/) contains expression and clinical prognosis data for more than 54,000 genes from the Gene Expression Omnibus (GEO), European Genome-phenome Archive (EGA), and TCGA cohorts and provides useful tools to analyze the effect of queried genes on cancer patient prognosis (17). The PrognoScan database (http://dna00.bio.kyutech.ac.jp/PrognoScan/) is an online platform used to analyze the prognostic value of queried genes in publicly available microarray datasets across 13 types of cancer (18).

### Genetic Alteration Analysis

The genetic mutation of SPINT1/2 and its correlation with patient survival were investigated using the cBioportal (http://www.cbioportal.org/). The cBioportal database integrates multidimensional cancer genomics data with interactive analyzing modules for research on gene alteration, co-expression profiles, survival, and pathways (19). Moreover, the Catalogue Of Somatic Mutations In Cancer (COSMIC) database (https://cancer.sanger.ac.uk/cosmic/) was used to mine the distributions of genetic alteration of SPINT1/2. The COSMIC database contains comprehensive somatic mutation data of human cancers and offers access to genetic alteration profiles in different contexts (20, 21).

### Methylation Analysis

The methylation differences between SPINT1/2 promoters and gene bodies were investigated using DiseaseMeth 2.0 (http://bio-bigdata.hrbmu.edu.cn/diseasemeth/). This database provides direct access to high-throughput methylome data of 679,602 samples and visualization tools (22). The relationship between methylation and expression of SPINT1/2 was examined using the cBioportal database. We looked into the prognostic values of methylated sites in breast cancer with MethSurv (https://biit.cs.ut.ee/methsurv/), and the ‘single CPG’ module was used to draw the survival plots and violin plots. The MethSurv database uses DNA methylation data from the Genome Data Analysis Center Firehose (http://gdac.broadinstitute.org/) across 25 types of cancers and provides mining solutions to facilitate methylation studies (23).

### Identification of Co-Expressed Genes and Enrichment Analysis

Using the LinkedOmics database (http://www.linkedomics.org/) (24), we screened the co-expressed genes of SPINT1/2 in TCGA breast cancer RNA-seq data. Pearson correlation test was applied, and the top 500 correlated genes (ranked by the absolute value of the correlation score) of SPINT1 and SPINT2 were identified. LinkInterpreter module and Gene Set Enrichment Analysis (GSEA) were used to investigate the enriched biological process (BP) and KEGG pathways. Additionally, common co-expressed genes were obtained by cross-referencing the respective top 500 co-expressed genes of SPINT1/2, and the protein–protein interaction (PPI) network of the common co-expressed genes was constructed with String database (https://string-db.org/) (25). The CluGO plugin in Cytoscape v3.8.2 was employed to perform the BP and KEGG pathway enrichment analyses of the common co-expressed genes. Core nodes and hub genes of the PPI network were identified with Cytoscape plugins MCODE and cytoHubba, respectively. The selection criteria in MCODE were as follows: MCODE score >5 points, degree cut-off = 2, node score cut-off = 0.2, Max depth = 100, and k-Score = 2.

### Statistical Analysis

Inclusion criteria for Oncomine datasets were set as follows: *P <*1E−4, fold change higher or lesser than 2, and gene rank of the top 10%. The student’s t-test was applied to compare the expression differences in Oncomine datasets. The Wilcoxon signed-rank test was performed to assess the expression of SPINT1/2 and hub genes in normal, and breast cancer tissues with R 3.6.3. For comparisons between two groups in the Breast Cancer Gene-Expression Miner v4.5 analysis, student’s t-test was applied, and one-way ANOVA followed by Dunnett’s multiple comparisons was performed when three groups were compared. The log-rank test was conducted for *P*-value in Kaplan–Meier plotter and cBioportal. The Cox *P* values were presented in PrognoScan. In the survival plots of MethSurv, a likelihood-ratio test was applied. Pearson’s correlation coefficient was used to measure the linear dependence between variables in the cBioportal and LinkedOmics. *P <*0.05 was considered to be statistically significant (*, *P* <.05; **, *P* <.01; ***, *P* <.001).

## Results

### Expression of SPINT1 and SPINT2 in Breast Cancer

**Figure 1:**
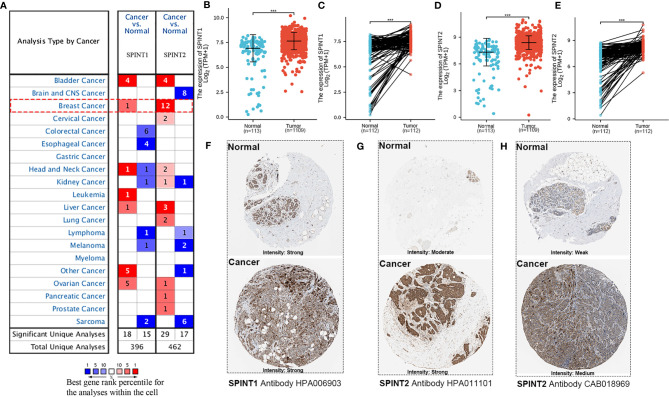
SPINT1/2 were overexpressed in breast cancer. **(A)** datasets reporting the upregulated expression of SPINT1/2 in Oncomine. The differential expression of SPINT1 mRNA in non- **(B)** and paired **(C)** breast cancer tissues. The mRNA expression of SPINT2 in non- **(D)** and paired **(E)** breast cancer samples. The protein expression of SPINT1 **(F)** and SPINT2 **(G, H)** in normal and cancer tissues from HPA database. *P <.05; **P <.01; ***P <.001.

First, we analyzed SPINT1/2 mRNA expression in normal and breast cancer tissues using the Oncomine database. Results showed one out of 45 datasets for SPINT1 and 12 out of 53 analyzes for SPINT2 reported mRNA upregulation in breast cancer tissues (**Figure 1A**). The complete transcriptional profiles of SPINT1/2 are shown in **Table S1**. Moreover, analysis of the RNA-Seq data of 1,222 TCGA breast cancer samples showed that SPINT1/2 were overexpressed in non- and paired cancer tissues compared to normal controls (**Figures 1B–E**). To further evaluate SPINT1/2 protein expression in normal versus breast cancer tissues, we explored the HPA database. The results showed the high intensity of SPINT1 staining in normal and cancer tissues (**Figure 1F**). Meanwhile, the SPINT2 intensity was medium in normal and robust in malignant samples, respectively, when incubated with the HPA006903 antibody (**Figure 1G**). Moreover, the normal tissues showed weak staining, but breast cancer tissues were moderately stained with CAB018969 antibody incubation (**Figure 1H**).

### Correlation Between SPINT1/2 Expression and Molecular Subtypes

**Figure 2:**
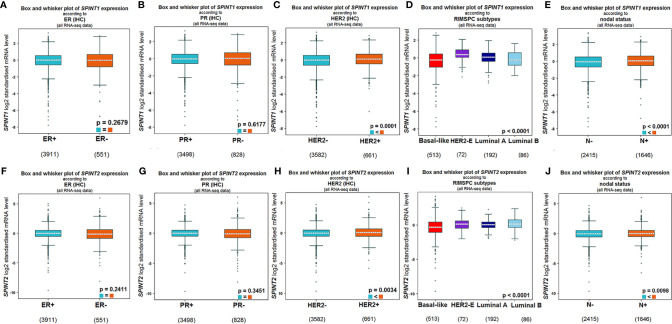
SPINT1/2 expression correlated with HER2 and node status. Relationship between SPINT1 expression with ER **(A)**, PR **(B)**, HER2 **(C)**, molecular subtypes **(D)** and node status **(E)**. Relationship between SPINT2 expression with ER **(F)**, PR **(G)**, HER2 **(H)**, molecular subtypes **(I)** and node status **(J)** from bc-GenExMiner v4.5.

We explored the bc-GenExMiner v4.5 for the relationship between SPINT1/2 expression and molecular subtypes of breast cancer. In this analysis, RNA-seq data from TCGA and Sweden Cancerome Analysis Network-Breast database (SCAN-B) were selected. The results showed that SPINT1 expression did not correlate with the status of estrogen receptor (ER) (*P* = 0.2679) or progesterone receptor (PR) (*P* = 0.6177) (**Figures 2A, B**), but was significantly related to HER2 status. Patients with positive HER2 lesions had a higher expression of SPINT1 (*P* = 0.0001) (**Figure 2C**). HER2-expression patients showed the highest SPINT1 level, and the basal-like group presented the lowest abundance (*P <*0.0001) (**Figure 2D**). Moreover, SPINT1 expression was significantly correlated with node status, and patients with node involvement had a relatively higher SPINT1 expression (*P <*0.0001) (**Figure 2E**).

In terms of SPINT2, its expression was not related to ER (*P* = 0.2411) or PR (*P* = 0.3451) status (**Figures 2F, G**), but was significantly correlated with HER2 status. SPINT2 expression was higher in HER2 positive versus negative patients (*P* = 0.0034) (**Figure 2H**). Likewise, the HER2 expression group showed the highest expression of SPINT2, and the basal-like group had the lowest abundance (*P <*0.0001) (**Figure 2I**). In addition, SPINT2 expression was higher in node-positive patients than in node-free patients (*P* = 0.0098) (**Figure 2J**).

### Elevated SPINT1/2 Expression in HER2+ Breast Cancer

**Figure 3:**
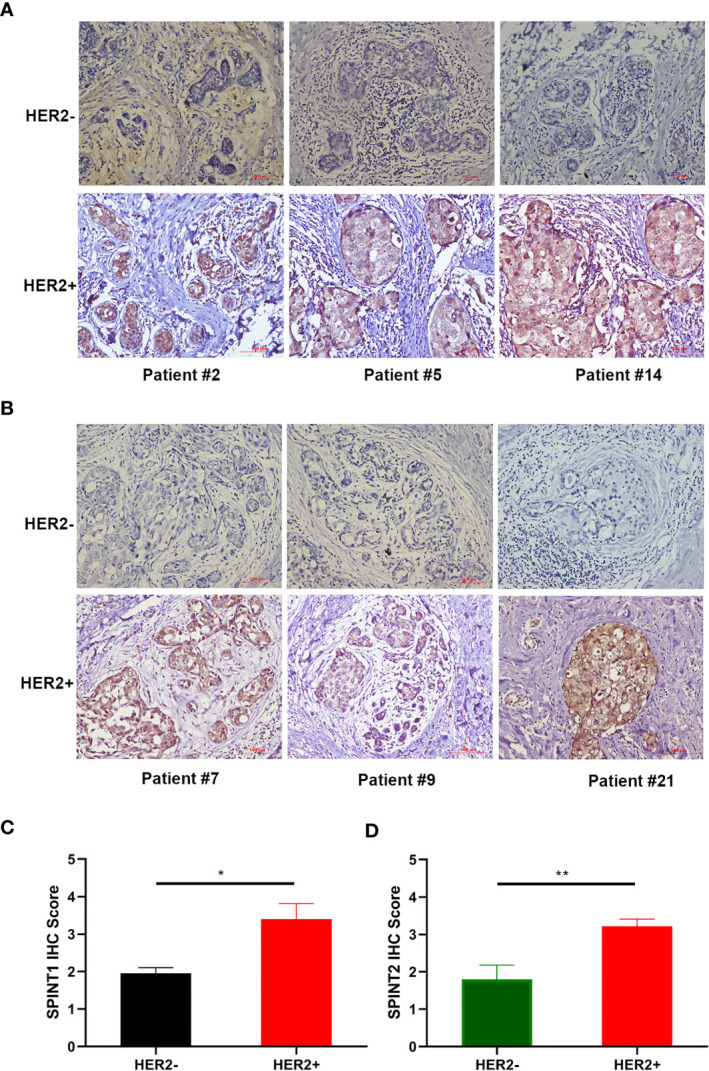
Protein expression of SPINT1/2 in HER2+ breast cancer. Representative images of SPINT1 **(A)** and SPINT2 **(B)** expression in cancer tissues. Semiquantitative results of SPINT1 **(C)** and **(D)** expression in HER2± breast cancer. *P < .05 and **P < .01.

As SPINT1/2 mRNA expression were found to be upregulated in HER2+ breast cancer, we further appraised their protein level in 21 breast cancer specimens *via* IHC. Representative images are presented in **Figures 3A, B**. Semiquantitative analyses revealed that SPINT1/2 expression were significantly increased in HER2+ breast cancer tissues than that in HER2- samples (*P* <0.05) (**Figures 3C, D**). These data suggested an upregulated expression of SPINT1/2 in HER2+ breast cancer, which was consistent with the bioinformatic analyses and substantiated the correlation between the status of HER2 and SPINT1/2 expression.

### SPINT1/2 Expression Correlated With Prognosis in Breast Cancer Patients

**Figure 4:**
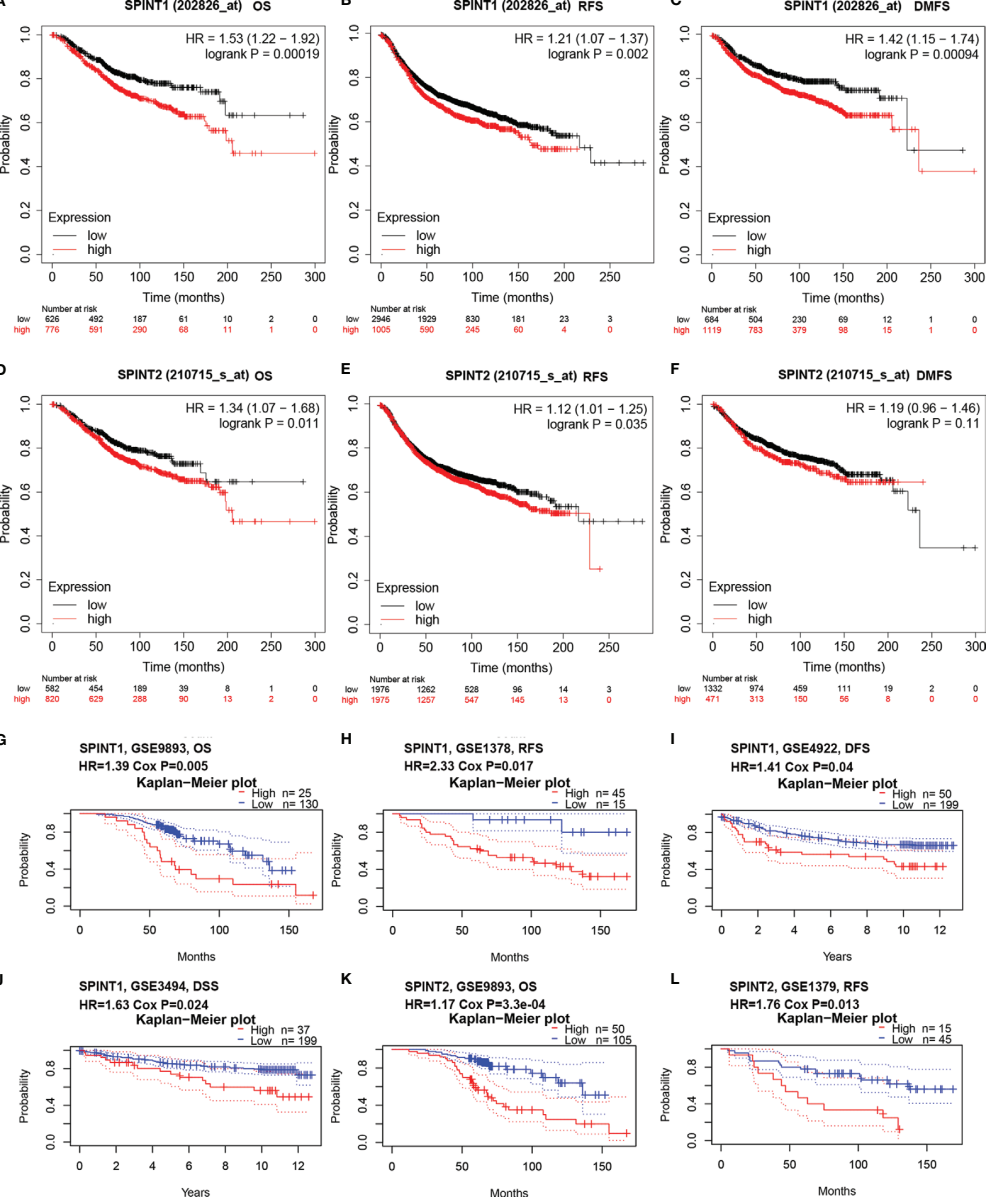
SPINT1/2 were correlated with patient prognosis. Correlation between SPINT1 expression and OS **(A)**, RFS **(B)** and DMFS **(C)** from Kaplan–Meier plotter. Association between SPINT2 expression and OS **(D)**, RFS **(E)** and DMFS **(F)** Kaplan–Meier plotter. The correlation between SPINT1 expression and the OS from GSE9893 **(G)**, RFS from GSE1378 **(H)**, DFS from GSE4922 **(I)** and DSS from GSE3494 **(J)**. The correlation between SPINT2 expression and the OS from GSE9893 **(K)** and the RFS from GSE1379 **(L)** on PrognoScan server.

We employed Kaplan–Meier plotter to examine the correlation between SPINT1/2 expression and patient prognosis, specifically overall survival (OS), relapse-free survival (RFS)), and disease metastasis-free survival (DMFS). The results showed that higher expression of SPINT1 was correlated with poorer OS (HR = 1.53, 95% CI = 1.22–1.92, *P* = 0.00019), RFS (HR = 1.21, 95% CI = 1.07–1.37, *P* = 0.002), and DMFS (HR = 1.42, 95% CI = 1.15–1.74, *P* = 0.00094) in breast cancer patients (**Figures 4A–C**). Similarly, the elevated expression of SPINT2 was related to worse OS (HR = 1.34, 95% CI = 1.07–1.68, *P* = 0.011) and RFS (HR = 1.12, 95% CI = 1.01–1.25, *P* = 0.035) (**Figures 4D, E**). However, there was no significant impact of SPINT2 on DMFS (HR = 1.19, 95% CI = 0.95–1.46, *P* = 0.11) (**Figure 4F**).

We further investigated the prognostic correlations of SPINT1/2 in breast cancer using the PrognoScan database. The results of two independent microarray datasets GSE9893 and GSE1378, respectively, showed that the upregulated expression of SPINT1 resulted in poorer OS (HR = 1.39, Cox *P* = 0.005) and RFS (HR = 2.33, Cox *P* = 0.017) (**Figures 4G, H**). In addition, the results of the GSE4922 and GSE3494 cohorts showed significant correlations between SPINT1 expression and disease-free survival (DFS) (HR = 1.14, Cox *P* = 0.04) and disease-specific survival (DSS) (HR = 1.63, Cox *P* = 0.024) (**Figures 4I, J**). Meanwhile, the elevated expression of SPINT2 was found to be correlated with a worse OS (HR = 1.17, Cox *P* = 3.3e−04) and RFS (HR = 1.76, Cox *P* = 0.013) in breast cancer (**Figures 4K, L**). These findings indicate that SPINT1 and SPINT2 are valuable biomarkers for prognosis in breast cancer patients.

### Correlation Between SPINT1/2 Expression and Clinicopathological Characteristics in Breast Cancer

Using Kaplan–Meier plotter, we also examined the correlation between SPINT1/2 expression and patient prognosis with restricted clinicopathological characteristics. The results showed that SPINT1 was significantly correlated with OS, RFS, and DMFS and with the TP53 status and molecular subtypes, except luminal A. Higher SPINT1 expression was correlated with poorer prognosis in ER+ and ER− subgroups. The expression of SPINT1 was significantly associated with DMFS irrespective of ER status, molecular subtypes, or grades (**Table 1**). Moreover, the highest HR was detected in the correlation between SPINT1 expression and DMFS in HER2+ patients (HR = 7.74, *P* = 0.0011), suggesting that SPINT1 influenced patient clinical outcomes, possibly by affecting tumor metastasis, particularly in the HER2+ subgroup.

**Table 1:** The correlation between SPINT1 expression and prognosis in breast cancer patients with different clinicopathological parameters.

Clinicopathological characteristics	OS (n = 1,402)			RFS (n = 3,951)			DMFS (n = 1,803)		
	N	Hazard ratio	*P* value	N	Hazard ratio	*P* value	N	Hazard ratio	*P* value
**ER status**									
ER positive	548	1.51 (1–2.28)	0.048	2,061	0.94 (0.8–1.11)	0.4582	664	1.63 (1.16–2.29)	0.0049
ER negative	251	1.73 (1.1–2.74)	0.0173	801	1.26 (0.99–1.59)	0.0588	275	2.12 (1.35–3.34)	9e-04
**PR status**									
PR positive	83	0.22 (0.05–0.88)	0.0193	589	1.48 (0.98–2.24)	0.0602	191	0.48 (0.21–1.11)	0.0796
PR negative	89	0.51 (0.15–1.75)	0.2721	549	1.2 (0.9–1.61)	0.216	154	1.99 (0.93–4.28)	0.0711
**HER2 Status**									
HER2 positive	129	0.53 (0.27–1.07)	0.072	252	0.7 (0.42–1.17)	0.1665	126	0.59 (0.26–1.34)	0.2048
HER2 negative	130	0.58 (0.24–1.37)	0.2048	800	0.77 (0.58–1.01)	0.054	150	1.6 (0.65–3.97)	0.3054
**Intrinsic type**									
Luminal A	611	1.39 (0.97–1.99)	0.745	1,933	1.2 (1–1.45)	0.0543	968	1.6 (1.18–2.16)	0.0024
Luminal B	433	2.31 (1.47–3.63)	0.0002	1,149	1.34 (1.09–1.66)	0.0062	449	1.74 (1.14–2.67)	0.0093
HER2+	117	2.54 (1.66–5.55)	0.0157	251	1.71 (1.14–2.55)	0.0082	125	7.74 (1.8–30.98)	0.0011
Basal	241	1.82 (1.09–3.01)	0.0191	618	1.61 (1.25–2.07)	0.0002	261	2.45 (1.38–4.36)	0.0016
**Lymph node status**									
Lymph node positive	313	1.41 (0.95–2.09)	0.0826	1,133	0.76 (0.61–0.94)	0.0103	382	1.45 (0.9–2.34)	0.1261
Lymph node negative	594	1.75 (1.19–2.58)	0.0042	2,020	1.15 (0.97–1.36)	0.1186	988	1.39 (1.05–1.85)	0.0227
**Grade**									
1	161	0.62 (0.23–3.26)	0.3335	345	1.62 (0.96–2.72)	0.0683	188	2.07 (0.89–4.79)	0.0826
2	387	2.04 (1.28–3.26)	0.0022	901	1.43 (1.12–1.82)	0.0045	546	1.5 (1.4–2.17)	0.0297
3	503	1.76 (1.27–2.45)	0.0006	903	1.37 (1.1–1.71)	0.0042	458	2.28 (1.46–3.56)	0.0002
**TP53 status**									
Muted	111	2.66 (1.24–5.71)	0.0087	188	3.24 (1.55–6.78)	0.0009	83	3.04 (1.16–7.97)	0.0173
Wild type	130	1.53 (1.1–7.26)	0.0243	272	1.72 (1.05–2.8)	0.0293	150	1.6 (0.65–3.97)	0.0711

OS, overall survival; RFS, relapse-free survival; DMFS, disease metastasis-free survival.

TABLE 1 SPINT1 expression was correlated with prognosis in breast cancer patients with different characteristics from Kaplan–Meier plotter.

In addition, we found that SPINT2 expression was significantly correlated with OS, RFS, and DMFS in patients with ER+, luminal B, and grade 2 subgroups (**Table 2**). However, SPINT2 was only significantly associated with DMFS in the ER+, HER2-, luminal B, grade 2, and TP53 wild type groups, indicating that SPINT2 was possibly involved in metastasis in particular subtypes.

**Table 2:** The correlation between SPINT2 expression and prognosis in breast cancer patients with different clinicopathological parameters.

Clinicopathological characteristics	OS (n = 1,402)			RFS (n = 3,951)			DMFS (n = 1,803)		
	N	Hazard ratio	*P* value	N	Hazard ratio	*P* value	N	Hazard ratio	*P* value
**ER status**									
ER positive	548	1.47 (1.03–2.11)	0.033	2,061	1.25 (1.05–1.48)	0.0098	664	1.74 (1.16–2.63)	0.0072
ER negative	251	1.67 (0.99–2.82)	0.05	801	1.21 (0.97–1.52)	0.093	275	1.42 (0.92–2.18)	0.1093
**PR ststus**									
PR positive	83	4.16 (0.86–20.11)	0.055	589	1.29 (0.89–1.86)	0.1793	192	2.13 (0.87–5.19)	0.0887
PR negative	89	3.05 (0.88–10.54)	0.0637	549	0.82 (0.6–1.13)	0.2276	192	1.58 (0.87–2.88)	0.1285
**HER2 Status**									
HER2 positive	129	2.25 (0.96–5.26)	0.0551	252	0.57 (0.36–0.9)	0.0135	126	1.54 (0.79–3.01)	0.2042
HER2 negative	130	3.82 (1.57–9.28)	0.0015	800	1.27 (0.95–1.69)	0.1004	129	2.87 (1.05–7.84)	0.0314
**Intrinsic type**									
Luminal A	611	1.6 (1.13–2.29)	0.0078	1,933	1.22 (1.02–1.46)	0.027	918	1.35 (0.99–1.83)	0.057
Luminal B	433	1.51 (1.01–2.27)	0.0449	1,149	1.32 (1.08–1.62)	0.007	449	1.67 (1.15–2.43)	0.0063
HER2+	117	1.77 (0.9–3.48)	0.093	251	0.7 (0.45–1.08)	0.1035	125	0.62 (0.32–1.18)	0.1434
Basal	241	1.29 (0.77–2.17)	0.3286	618	1.2 (0.93–1.55)	0.1641	261	0.81 (0.48–1.36)	0.4301
**Lymph node status**									
Lymph node positive	311	1.69 (1.14–2.5)	0.0078	1,133	1.32 (1.08–1.61)	0.0059	382	0.66 (0.43–1.02)	0.0593
Lymph node negative	382	1.34 (0.86–2.09)	0.1986	2,020	1.15 (0.95–1.38)	0.1442	988	1.28 (0.96–1.71)	0.095
**Grade**									
1	161	2.19 (0.63–7.56)	0.2039	345	1.37 (0.78–2.41)	0.2768	188	1.73 (0.75–3.99)	0.1938
2	387	1.77 (1.15–2.73)	0.0086	901	1.45 (1.14–1.86)	0.0028	546	1.88 (1.31–2.69)	0.0005
3	503	1.54 (1.05–2.26)	0.0271	903	1.15 (0.92–1.44)	0.2068	458	1.38 (0.94–2.01)	0.0956
**TP53 status**									
Muted	111	1.64 (0.73–3.65)	0.2231	188	1.79 (1.09–2.92)	0.019	83	2.37 (0.91–6.17)	0.0695
Wild type	187	1.77 (0.92–3.41)	0.0854	273	1.74 (1.13–2.68)	0.0114	109	2.56 (1.18–5.56)	0.0137

OS, overall survival; RFS, relapse-free survival; DMFS, disease metastasis-free survival.

TABLE 2 SPINT2 expression was associated with prognosis in breast cancer patients with different characteristics.

### Genetic Alteration Frequency of SPINT1/2 Was Low and Not Related to the Prognosis

**Figure 5:**
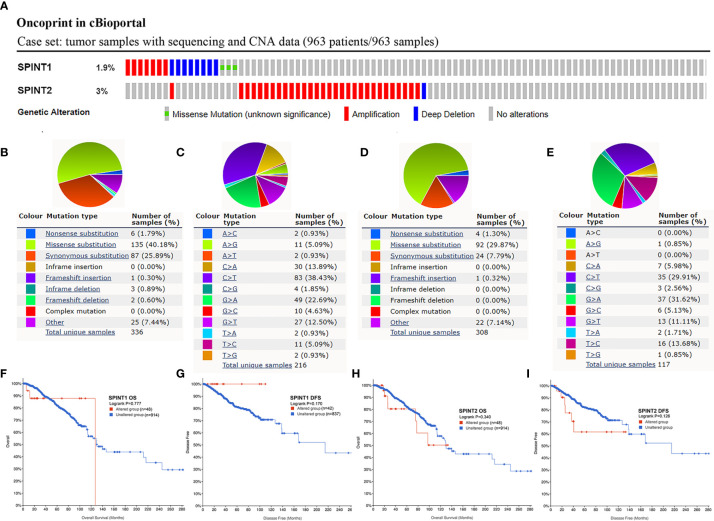
Genetic alteration frequency of SPINT1/2 was low and not related to prognosis. Genetic mutations in SPINT1/2 **(A)**, mutation subtypes distribution of SPINT1 **(B)**, nucleotide changes in SPINT1 **(C)**. Alteration subtypes of SPINT2 **(D)**, nucleotide changes of SPINT2 **(E)**. The correlation between SPINT1 gene alteration and OS **(F)** and DFS (**G**). The correlation between SPINT2 gene alteration and OS **(H)** and DFS **(I)**.

Mutations in protein-encoding genes induce expression changes in cancer (26), therefore, we investigated the genetic alteration of SPINT1/2 in breast cancer with the cBioportal database. The breast invasive carcinoma case set (TCGA, Firehose Legacy) containing 963 samples was selected, and the results showed that the mutation of SPINT1/2 was detected in 1.9% (18/963) and 3% (31/963) patients, respectively (**Figure 5A**). To unveil the mutation distributions of SPINT1/2 in breast carcinoma, we searched COSMIC database. The results showed that the alteration types of SPINT1 included nonsense substitution, missense substitution, synonymous substitution, and frameshift deletion. Missense substitution was the most common mutation for SPINT1, accounting for about 40% (**Figure 5B**). Moreover, various nucleotide changes in the substitution mutations were observed, of which C > T and G > A accounted for the largest proportion (**Figure 5C**). Similarly, missense substitutions, synonymous substitutions, and nonsense substitutions were detected in the SPINT2 mutation. The missense substitutions constituted the biggest percentage, which were approximately 29% of the 308 samples (**Figure 5D**). The nucleotide changes in observed mutations included C > A, C > T, C > G, G > A, and G > C. C > T was the most frequent change accounting for about 30% of the change (**Figure 5E**).

Subsequently, cBioportal was used to investigate the correlation between SPINT1/2 alteration and prognosis. Survival plots showed that SPINT1 genetic alteration was not significantly correlated with patient OS (log-rank *P* = 0.777) or DFS (log-rank *P* = 0.170) (**Figures 5F, G**). The mutation of SPINT2 had no impact on OS (log-rank *P* = 0.340) or DFS (log-rank P = 0.126) in breast cancer patients (**Figures 5H, I**).

### Methylation of SPINT1/2 Was Correlated With Prognosis in Breast Cancer

We explored the DiseaseMeth2.0 database for the SPINT1/2 DNA methylation status and found hypomethylation of SPINT1/2 in the promoter and the gene body (**Figures S1A–D**). The relationship between SPINT1/2 methylation and expression was further investigated using the cBioportal database. The results showed that SPINT1/2 methylation was negatively correlated with its expression in breast cancer (**Figures S1E, F**).

**Figure 6:**
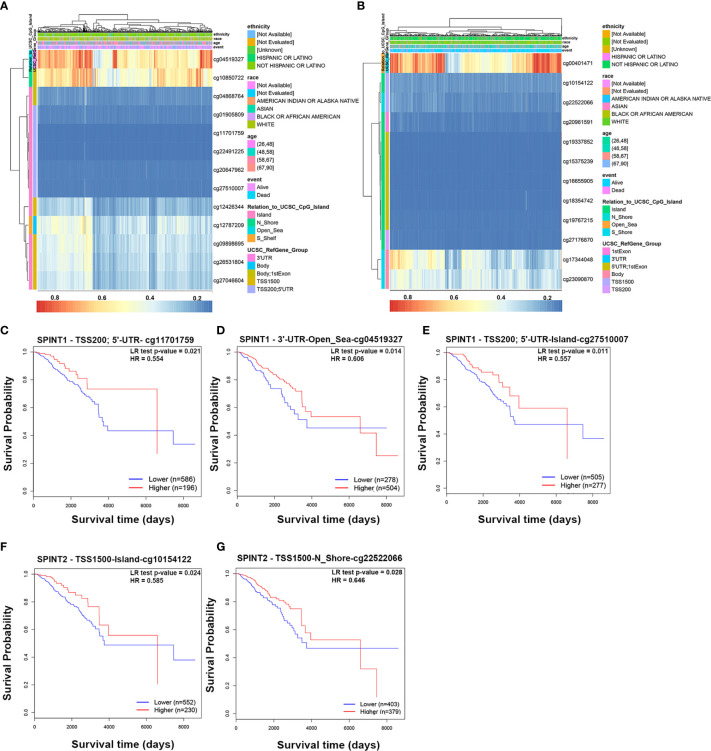
Methylation of SPINT1/2 was correlated with prognosis in breast cancer from MethSurv. Heatmaps of the association between methylation level of SPINT1 **(A)** and SPINT2 **(B)** and patient characteristics and genomic subregions. Association of methylation at TSS200; 5’UTR-cg11701759 **(C)**, 3’UTR-Open_Sea-cg04519327 **(D)** and TSS200; 5’UTR-Island-cg27510007 **(E)** in SPINT1 with patient OS. Correlation between methylation at TSS1500-Island-cg10154122 **(F)** and TSS1500-N_Shore-cg22522066 **(G)** in SPINT2 and OS.

Next, we used MethSurv to identify which methylation sites in SPINT1/2 were significantly correlated with breast cancer prognosis. According to the University of California Santa Cruz Genome Browser (UCSC) database, the CpG sites were grouped into six gene subregions: ‘TSS200’, ‘TSS1500’, ‘first exon’, ‘5’ UTR’, ‘body’ and ‘3’ UTR’ (23). The heat maps evaluating the relationship of SPINT1/2 methylation levels with the available patient characteristics and gene subregions were plotted with ‘Gene Visualization’ (**Figures 6A, B**). By analyzing all 13 methylation sites of SPINT1 in breast cancer patients, we found three hypomethylated sites (TSS200; 5’-UTR-cg11701759, 3’-UTR-Open_Sea-cg04519327, and TSS200; 5’-UTR-Island-cg27510007) were significantly correlated with a poorer OS (**Figures 6C–E**). Of the 12 methylation sites in SPINT2, only two hypomethylated sites (TSS1500-Island-cg10154122 and TS1500-N_Shore-cg22522066) were associated with poorer OS (**Figures 6F, G**).

### Identification and Enrichment Analysis of SPINT1/2 Co-Expressed Genes

**Figure 7:**
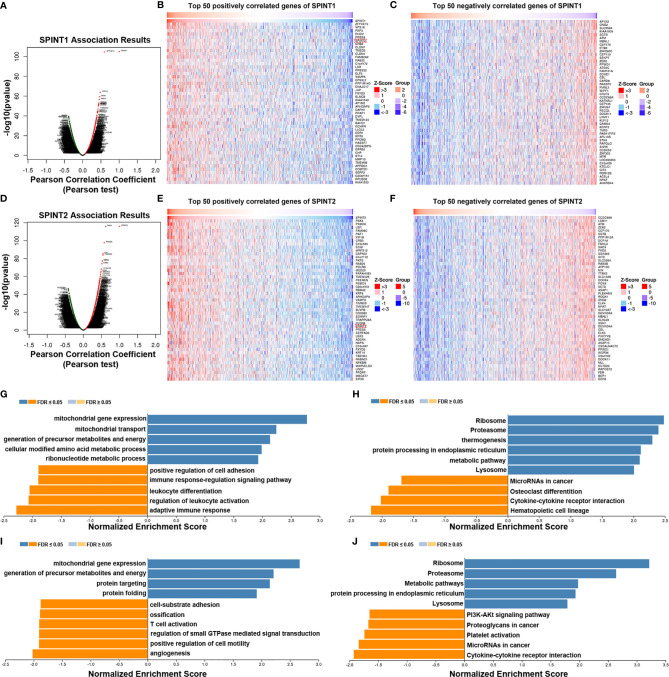
Screening and enrichment analysis of SPINT1/2 co-expressed genes by Linkedomics. Volcano plot of SPINT1 co-expressed genes **(A)**. Top 50 positively **(B)** and top 50 negatively **(C)** correlated genes of SPINT1. Volcano plot of SPINT2 co-expressed genes **(D)**. Top 50 positively **(E)** and top 50 negatively **(F)** correlated genes of SPINT2. Biological process **(G)** and KEGG pathway **(H)** enrichment of the SPINT1 co-expressed genes. Biological process **(I)** and KEGG pathway **(J)** enrichment of the SPINT2 co-expressed genes.

The Linkedomics database was used to identify co-expressed genes of SPINT1 and SPINT2 in TCGA. The results showed that 3,499 genes (red dots) were positively and 6,420 genes (green dots) were negatively correlated with SPINT1 in breast cancer (FDR <0.01) (**Figure 7A** and **Table S2: Sheet 1**). Of note, SPINT2 (red rectangle) was among the top 50 positively correlated SPINT1 genes (**Figure 7B**). The top 50 negatively correlated genes of SPINT1 are shown in **Figure 7C**. Meanwhile, 4,411 genes (red dots) were positively, and 6,545 genes (green dots) were negatively associated with SPINT2 in breast cancer (FDR <0.01) (**Figure 7D** and **Table S2: Sheet 2**). SPINT1 was in the top 50 positively correlated genes of SPINT2 (**Figure 7E**), and the top 50 negatively correlated genes are shown in **Figure 7F**.

We investigated the functions of SPINT1/2 co-expressed genes using the LinkedOmics LinkInterpreter module. GSEA was applied for GO BP and KEGG enrichment of the respective correlated genes of SPINT1 and SPINT2. The results showed that the SPINT1 co-expressed genes were involved in mitochondrial gene expression, mitochondrial transport, precursor metabolites, and energy generation of precursor metabolites and energy. Moreover, BP terms, such as positive regulation of cell adhesion and immune responses were negatively enriched (**Figure 7G**). KEGG pathway enrichment revealed that the co-expressed genes were involved in the ribosome, proteasome, endoplasmic reticulum protein processing, and metabolic signaling (**Figure 7H**). Meanwhile, the SPINT2 co-expressed genes were primarily involved in mitochondrial gene expression, the generation of precursor metabolites and energy, protein targeting, and protein folding (**Figure 7I**). Notably, biological processes, such as cell-substrate adhesion and T-cell activation are inversely regulated. KEGG pathway enrichment indicated that the co-expressed genes of SPINT2 played a role in signaling the ribosome, proteasome, metabolic pathways, and protein processing in the endoplasmic reticulum (**Figure 7J**).

### Enrichment Analysis and PPI Network of the Common Co-Expressed Genes

**Figure 8:**
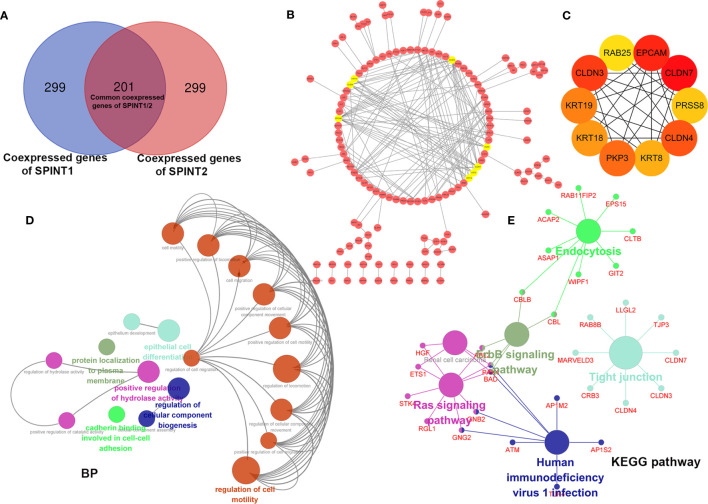
Enrichment analysis and PPI network construction of SPINT1/2 common co-expressed genes with Cytoscape. Cross-reference of respective top 500 co-expressed genes of SPINT1/2 **(A)**, PPI network of SPINT1/2 common co-expressed genes **(B)** and hub genes **(C)** in the PPI network. BP terms **(D)** and KEGG pathways **(E)** enriched of SPINT1/2 common co-expressed genes.

The Linkedomics results showed that SPINT1 and SPINT2 were reciprocally correlated in breast cancer (Pearson correlation = 0.437, *P* = 2.103e−52) (**Figure S1G**), which was close to the correlation statistics from cBioportal (TCGA, Firehose Legacy) (Pearson correlation = 0.42, *P* = 4.68e−83) (**Figure S1H**). To further investigate the roles SPINT1 and SPINT2 jointly played in breast cancer, the common co-expressed genes were screened first by cross-referencing the respective top 500 correlated genes of SPINT1 and SPINT2 (ranking by the absolute value of Pearson correlation) (**Table S3: Sheet 1, Sheet 2**), and a total of 201 common co-expressed genes were obtained (**Figure 8A** and **Table S4**).

We further constructed the PPI network of the 201 common co-expressed genes using the String database. The results were then imported to Cytoscape and refined after removing disconnected nodes. Using the MCODE plugin, the top eight most important modules, including PKP3, KRT8, KRT18, KRT19, CLDN3, CLDN4, CLDN7, and EPCAM (amber circles) were screened (**Figure 8B**). We filtered out the top 10 hub genes in the PPI network based on the MCC scores with the CytoHubba plugin, they were CLDN7, CLDN3, CLDN4, EPCAM, PKP3, KRT19, KRT8, KRT18, PRSS8, and RAB25 (**Figure 8C** and **Table S5**).

Subsequently, we investigated the biological clustering of the common co-expressed genes of SPINT1/2 using ClueGO in Cytoscape for BP and KEGG enrichment. ClueGO incorporates GO terms and KEGG pathways and creates functionally organized GO/pathway term networks (27). In this analysis, medium network specificity and yFiles Radial Layout were applied, and other parameters were default. The results showed that 19 terms (circles in various colors) including regulation of cell migration, cadherin binding involved in cell–cell adhesion, and regulation of cellular component biogenesis were significantly enriched (*P <*0.05) (**Figure 8D**). KEGG pathway enrichment indicated that these common co-expressed genes were involved in tight junction, Ras signaling pathway, erbB signaling pathway, endocytosis, and human immunodeficiency virus 1 infection (**Figure 8E**). These results suggest that SPINT1 and SPINT2 may be jointly responsible for cell adhesion in breast cancer.

### Expression and Prognostic Significance of Hub Genes

**Figure 9:**
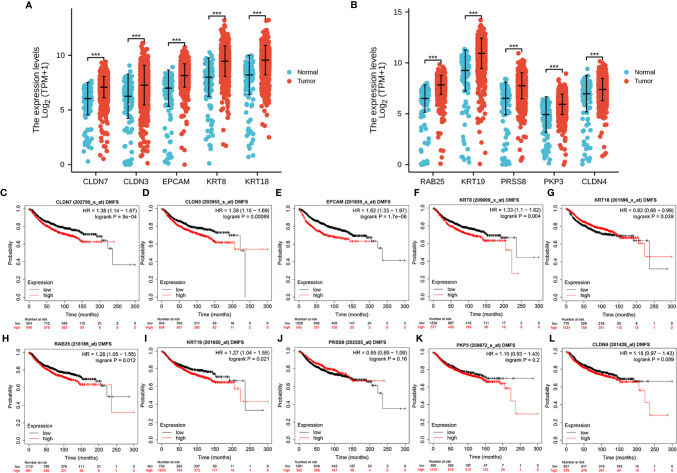
Expression and prognostic significance of the hub genes in TCGA. Differential expression of the 10 hub genes **(A, B)** in breast cancer. The correlation between patient DMFS and the expression of CLDN7 **(C)**, CLDN3 **(D)**, EPCAM **(E)**, KRT8 **(F)**, KRT18 **(G)**, RAB25 **(H)**, KRT19 **(I)**, PRSS8 **(J)**, PKP3 **(K)** and CLDN4 **(L)**. ***P < .001.

We examined the differential expression of the hub genes in breast cancer patients from TCGA cohorts. The results showed that all 10 hub genes were overexpressed in cancer tissues (**Figures 9A, B**). We further analyzed the prognostic significance of the hub genes in breast cancer patients using Kaplan–Meier plotter and found that the upregulated expressions of CLDN7, CLDN3, EPCAM, KRT8, KRT18, RAB25, and KRT19 were significantly correlated with DMFS in breast cancer (**Figures 9C–I**), but PRSS8, PKP3, and CLDN4 expression was not (**Figures 9J–L**).

Additionally, we investigated the correlation between the expression of 10 hug genes and the OS in breast cancer. The results showed that higher levels of CLDN4, CLDN7, CLDN3, EPCAM, PKP3, KRT8, and RAB25 were correlated with the poorer OS (**Figures S2A–G**). However, this correlation was not found for KRT18, KRT19, and PRSS8 (**Figures S2H–J**).

## Discussion

Although most studies involving SPINT1 or SPINT2 reported reduced expression in cancers (28–32), we observed a paradoxical upregulated expression of SPINT1/2 in breast cancer. Due to the high heterogeneity, the histopathological characteristics and clinical manifestations of breast cancer are subtype-dependent. Accordingly, we discovered that SPINT1/2 were significantly related to HER2 status and node status, but not to ER or PR status. Specifically, patients with HER2+ and node involvement had a relatively higher expression of SPINT1/2 than node-free patients.

We found that SPINT1/2 expression was significantly correlated with prognosis in breast cancer. SPINT1 was correlated with OS, RFS, and DMFS in patients with different molecular subtypes, except for luminal A. The highest HR was observed in the correlation between SPINT1 expression and DMFS in HER2+ patients. HER2, a transmembrane tyrosine kinase receptor, is considered a strong predictive biomarker of regional and distant metastasis, leading to an increased malignancy and poor prognosis (33, 34). These results suggest that SPINT1 may influence patient clinical outcomes by facilitating tumor dissemination. Targeting SPINT1 may be a promising strategy for breast cancer patients, particularly for the HER2+ subgroup.

Besides, SPINT2 upregulation was correlated with unfavorable OS and RFS in breast cancer. It was, however, not related to the DMFS. However, in the subgroup analysis, SPINT2 was correlated with poorer DMFS in particular types, specifically in patients with luminal A, luminal B, and HER2−, which implied that SPINT2 was possibly involved in metastasis in particular subtypes, thus rendering adverse outcomes in breast cancer patients.

Although genetic alterations were observed in SPINT1/2, they were less frequent and did not affect patient prognosis. SPINT2 has been reported to be hypermethylated in many other cancers (28, 35). However, we found decreased DNA methylation of SPINT2 in breast cancer. Additionally, SPINT1 methylation is decreased in breast cancer, representing the same status as in hepatocellular carcinoma (36). Moreover, several hypomethylated sites in the SPINT1/2 genes correlated with patient prognosis have also been identified, representing ideal aberrantly demethylated sites of SPINT1/2 in breast cancer.

To further investigate the independent and combined functions of SPINT1 and SPINT2, we screened and conducted enrichment analysis of their respective co-expressed genes and common co-expressed genes. We discovered that SPINT1 and SPINT2 have different roles and overlapping functions in breast cancer biology. In our study, some BP and KEGG pathways modulated by SPINT1 or SPINT2 were not enriched by the other. Previous studies also identified that their functions in matriptase trafficking were cell-type dependent (37–40), and their immunoreactivity locations were different (7, 41). Meanwhile, SPINT1 and SPINT2 are well-documented upstream HGF precursors regulators and modulate epithelial integrity (42, 43). Their common co-expressed genes were jointly involved in regulating cell migration, cadherin binding in cell–cell adhesion, and cellular component biogenesis regulation. Moreover, in the KEGG enrichment analysis, two metastasis-related pathways, Ras signaling and erbB signaling were significantly enriched. Ras signaling is a crucial determinant of breast cancer distant dissemination and positively correlated with HER2+ subtypes (44, 45). erbB signaling is widely involved in regulating breast cancer cell proliferation, epithelial-to-mesenchymal transition, metastasis, and drug resistance (46–48). More studies are needed to clarify the detailed direct relationship between SPINT1/2 and these pathways in the future.

SPINT1 and SPINT2 expression were reciprocally correlated in breast cancer, which was in accordance with reports that they were frequently co-expressed in the same cell (39, 41). By cross-referencing, 201 out of the top 500 co-expressed genes of SPINT1/2 were significantly correlated with both SPINT1 and SPINT2. We screened out eight core nodes and 10 hub genes in the PPI network of the common co-expressed genes, and the results showed that the core nodes and hub genes were primarily overlapped. CLDN7, CLDN3, and CLDN4, members of the claudin family, are integral membrane proteins of tight junctions (49). Dysregulation of the claudin family proteins plays an oncogenic role in some malignancies (50, 51). EPCAM is known for its role in preventing cell-cell adhesion, cell signaling, migration, proliferation, and differentiation (52). PKP3 is a member of the armadillo protein family, which plays a central role in tumorigenesis by regulating cell adhesion (53). Moreover, keratin families, such as KRT8, KRT18, and KRT19, were also screened. It is widely reported that the keratin family regulates intermediate filaments, which trigger cancer progression and metastasis (54, 55). All these results indicated that SPINT1 and SPINT2 jointly regulated cell attachment and metastasis in breast cancer.

## Data Availability Statement

The datasets presented in this study can be found in online repositories. The names of the repository/repositories and accession number(s) can be found in the article/**Supplementary Material**.

## Ethics Statement

This study was performed in line with the principles of the Declaration of Helsinki. The studies involving human participants were reviewed and approved by the Ethics Committee of Chongqing Medical University. The patients/participants provided their written informed consent to participate in this study.

## Author Contributions

SL and JLu conceived the project and reviewed the manuscript. QW and GY participated data analysis and wrote the manuscript. YZ, TA, JT, YJ, and JLe participated in discussion. All authors contributed to the article and approved the submitted version.

## Funding

This study was funded by National Natural Science Foundation of China, Grant/Award Numbers: 81772979 and 81472658.

## Conflict of Interest

The authors declare that the research was conducted in the absence of any commercial or financial relationships that could be construed as a potential conflict of interest.
