# Supplementary figures and images for: Comprehensive Analysis of the Expression and Prognostic Value of SPINT1/2 in Breast Carcinoma
Source: Front Endocrinol (Lausanne). 2021 Jul 26;12:665666. doi: 10.3389/fendo.2021.665666 (PMC8351597; doi:10.3389/fendo.2021.665666)

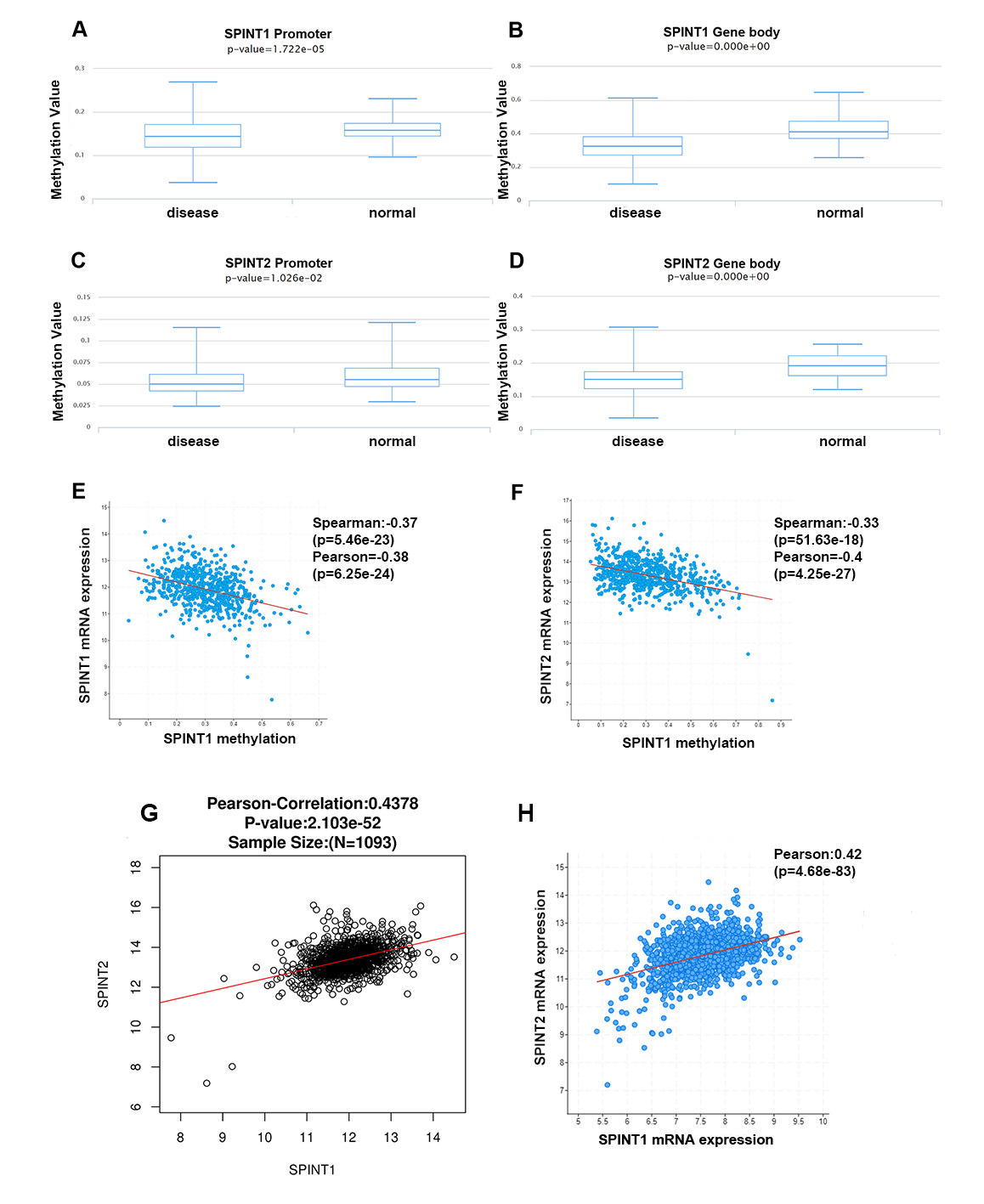

Supplement: Supplementary file 1 [file Image_1.tif]

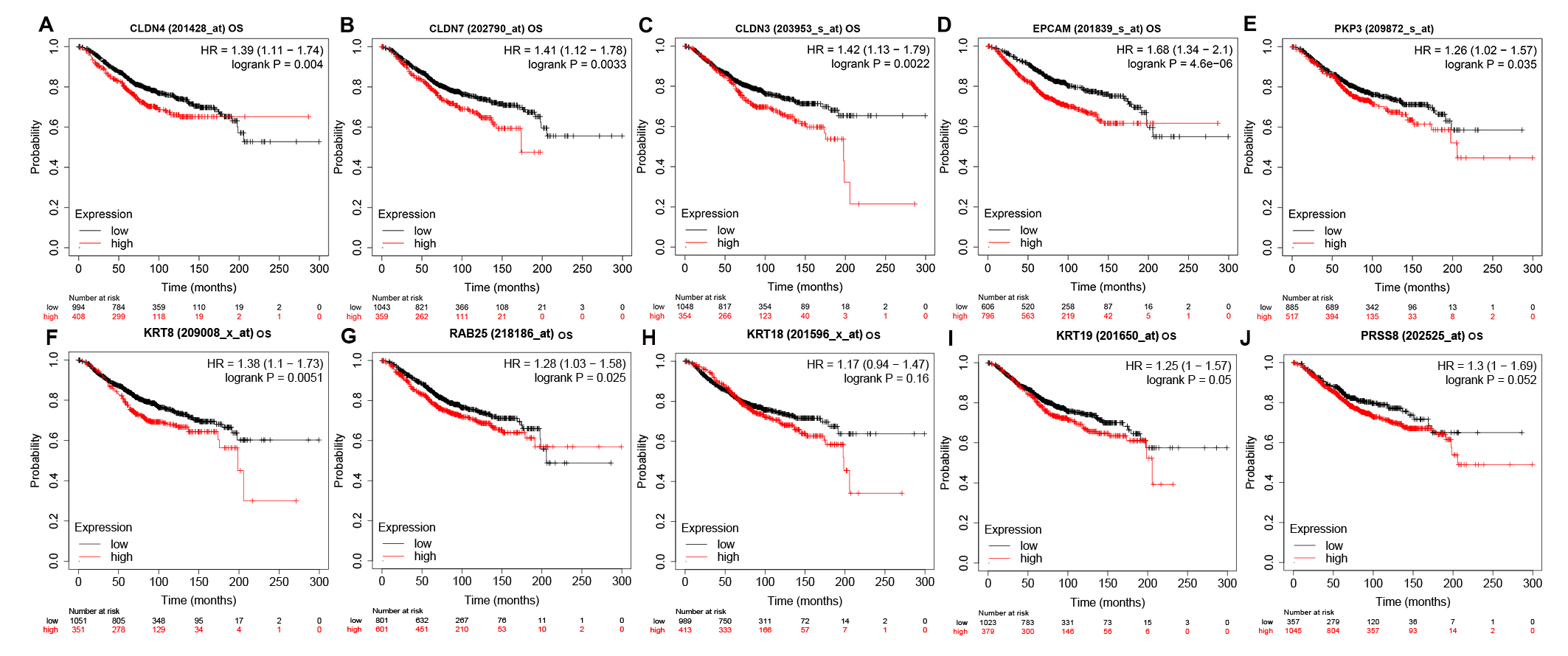

Supplement: Supplementary file 2 [file Image_2.tif]
